# The protective effects of Ninjin’yoeito against liver steatosis/fibrosis in a non-alcoholic steatohepatitis model mouse

**DOI:** 10.1007/s11418-024-01786-2

**Published:** 2024-03-18

**Authors:** Kyohei Takano, Marisa Kaneda, Yayoi Aoki, Nina Fujita, Shigeki Chiba, Seiwa Michihara, Li-Kun Han, Ryuji Takahashi

**Affiliations:** Kampo Research Laboratory, Pharmaceutical Company, Kracie, Ltd., 3-1 Kanebo-Machi, Takaoka, Toyama Japan

**Keywords:** Ninjin’yoeito, Steatosis, Fibrosis, Non-alcoholic steatohepatitis, Choline-deficient L-amino acid-defined high-fat diet

## Abstract

Non-alcoholic steatohepatitis (NASH) is a progressive fibrotic form of non-alcoholic fatty liver disease. Liver fibrosis leads to liver cancer and cirrhosis, and drug therapy for NASH remains lacking. Ninjin’yoeito (NYT) has shown antifibrotic effects in a model of liver fibrosis without steatosis but has not been studied for NASH. Therefore, we evaluated the efficacy of NYT in mice fed a choline-deficient, L-amino acid-defined, high-fat diet (CDAHFD) as a NASH model. Compared with the normal diet group, mice fed CDAHFD showed decreased body weight and increased white adipose tissue, liver weight, and triglyceride content in the liver. Furthermore, a substantial increase in the hepatic concentration of hydroxyproline, expression of α-smooth muscle actin (*α-SMA*), and transforming growth factor-β was observed in CDAHFD-fed mice. Masson’s trichrome and Picro-Sirius red staining revealed a remarkable increase in collagen fiber compared with the normal diet group. Compared with mice that received CDAHFD alone, those supplemented with NYT exhibited reduced hepatic triglyceride and hydroxyproline levels and *α-SMA* expression. Additionally, compared with the group fed CDAHFD alone, the stained liver tissues of NYT-treated mice exhibited a reduction in Masson’s trichrome- and Picro-Sirius red-positive areas. Locomotor activity was significantly reduced in the CDAHFD-fed group compared with the normal diet group. In the NYT-treated group, the CDAHFD-induced decrease in locomotor activity was significantly suppressed. The findings indicate that NYT inhibited fatty and fibrotic changes in the livers of NASH mice and alleviated the decrease in locomotor activity. Therefore, NYT may serve as a novel therapeutic approach for NASH.

**Graphical abstract:**

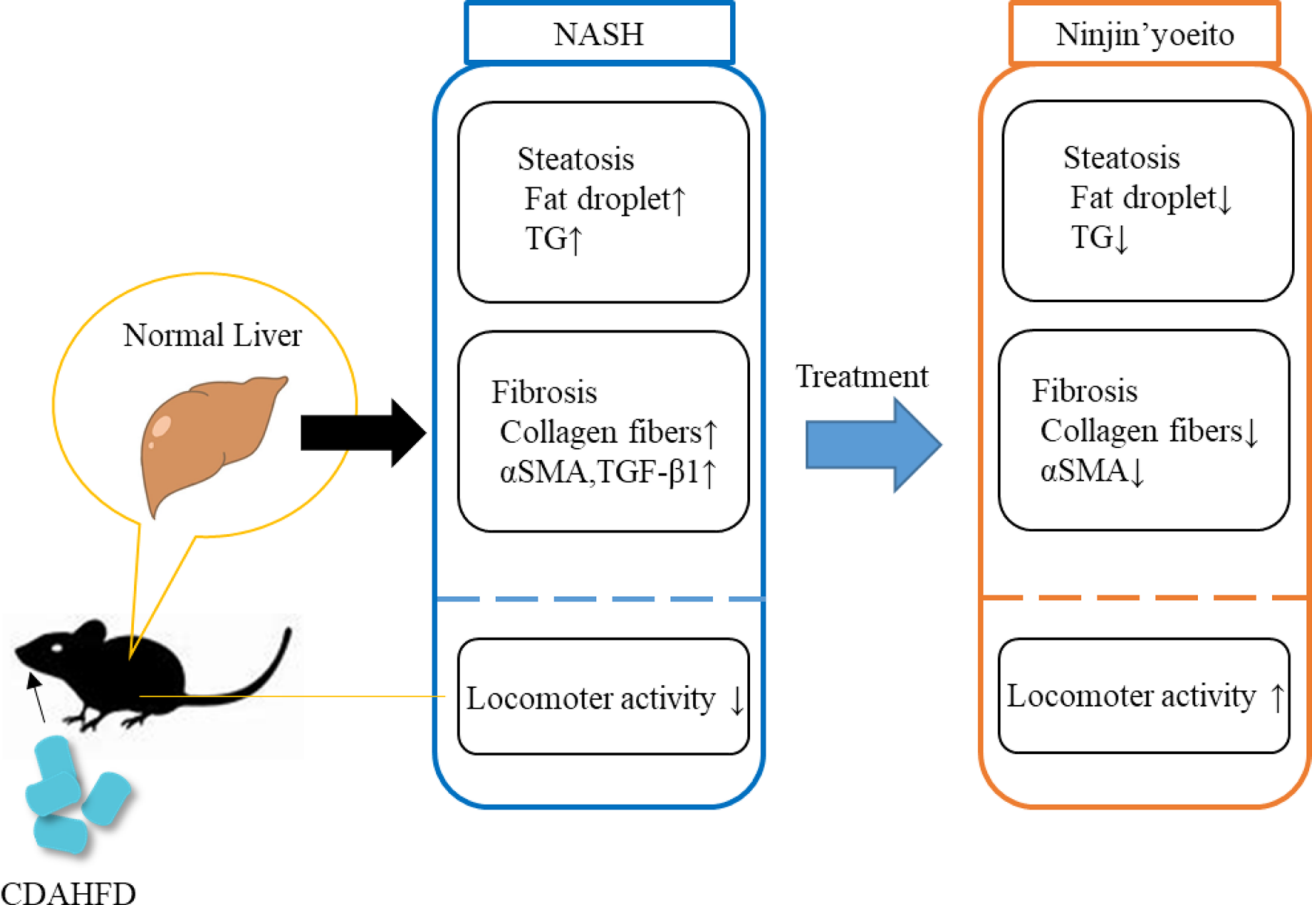

## Introduction

Non-alcoholic fatty liver disease (NAFLD) cases are increasing worldwide, with a prevalence rate of 20–25% in the adult population [1, 2]. Non-alcoholic steatohepatitis (NASH), a progressive fibrotic form of NAFLD, comprises 25% of all NAFLD diagnoses [1, 2]. NASH is characterized by liver fibrosis with fatty liver as a pathological finding. Fibrosis refers to a condition in which the extracellular matrix, mainly composed of collagen, is excessively deposited in an organ. As liver fibrosis poses a risk of cirrhosis and cancer, the progression of liver fibrosis is associated with an increase in mortality [3–5]. Therefore, suppressing the progression of fibrosis is critical to effectively treat NASH. A multiple parallel hit hypothesis [6] has been proposed for the onset of NASH, which states that NASH is caused by the simultaneous progression of steatosis and fibrosis.

Currently, the main treatments for NASH are lifestyle improvements such as weight loss and aerobic exercise, as effective drug treatment methods targeting the liver have not yet been established [7, 8]. Therefore, the development of drugs against fibrosis in NASH is very important. In recent years, Kampo formulations that are effective against liver steatosis have attracted attention, and studies have reported their antifibrotic effects [9]. Ninjin’yoeito (NYT) is composed of 12 substances—Ginseng, Rehmannia Root, Japanese Angelica Root, Atractylodes Rhizome, Poria Sclerotium, Cinnamon Bark, Polygala Root, Peony Root, Citrus Unshiu Peel, Astragalus Root, Glycyrrhiza, and Schisandra Fruit (Table 1) [10]—and is used to treat decreased physical strength post-illness and post-surgery, fatigue, malaise, anorexia, night sweats, cold limbs, and anemia [11–19]. Previous studies have reported that NYT suppresses hepatic collagen accumulation in liver fibrosis model rats [20]. However, the efficacy of NYT in NASH mice, model mice with liver steatosis, is unknown. Therefore, in this study, we aimed to examine the protective effect of NYT using mice with NASH that were fed a choline-deficient, L-amino acid-defined, high-fat diet (CDAHFD). In addition, chronic liver diseases such as NASH/NAFLD are accompanied by behavioral changes such as fatigue and depression [21–23]. In fact, fatigue has been reported as a primary symptom in patients with NASH/NAFLD [24, 25]. Given that several reports have used reduced locomotor activity as a surrogate marker for fatiguing behavior [26–28], we also evaluated on spontaneous locomotor activity in NASH mouse model.

**Table 1:** Composition (daily dose^a^) of Kampo drug Ninjin’yoeito (NYT)

Ingredients			Amount (g)
English name	Latin name	Original plant source and medicinal part	
Poria Sclerotium	*Poria*	The sclerotium of *Wolfiporia cocos* Ryvarden et Gilbertson (*Poria cocos* Wolf)	4
Japanese Angelica Root	*Angelicae Acutiloba Radix*	The root of *Angelica acutiloba* Kitagawa *or Angelica acutiloba* Kitagawa var. *sugiyamae* Hikino	4
Rehmannia Root	*Rehmanniae Radix*	The root of *Rehmannia glutinosa* Liboschitz var. *purpurea* Makino or *Rehmannia glutinosa* Liboschitz	4
Atractylodes Rhizome	*Atractylodis Rhizoma*	The rhizome of *Atractylodes japonica* Koidzumi ex Kitamura or *Atractylodes macrocephala* Koidzumi (*Atractylodes ovata* De Candolle)	4
Ginseng	*Ginseng radix*	The root of *Panax ginseng* C. A. Meyer (*Panax schinseng* Nees)	3
Cinnamon Bark	*Cinnamomi cortex*	The bark of the trunk of *Cinnamomum cassia* J. Presl	2.5
Citrus Unshiu Peel	*Citri Unshiu Pericarpium*	The pericarp of the ripe fruit of *Citrus unshiu* Marcowicz or *Citrus reticulata* Blanco	2
Polygala Root	*Polygalae Radix*	The root or root bark of *Polygala tenuifolia* Willdenow	2
Peony Root	*Paeoniae Radix*	The root of *Paeonia lactiflora* Pallas	2
Astragalus Root	*Astragali Radix*	The root of *Astragalus membranaceus* Bunge or *Astragalus mongholicus* Bunge	1.5
Schisandra Fruit	*Schisandrae Fructus*	The fruit of *Schisandra chinensis* Baillon	1
Glycyrrhiza	*Glycyrrhizae Radix*	The root and stolon of *Glycyrrhiza uralensis* Fischer or *Glycyrrhiza glabra* Linné	1

^a^Approximately, 6700 mg of water-dried extract of NYT was prepared in the GMP-standardized factory of Pharmaceutical Company, Kracie, Ltd. (Qingdao, China) with the composition described above

## Materials and methods

### NYT

NYT was manufactured by GMP Pharmaceutical Factory of Pharmaceutical Company, Kracie, Ltd. (lot no. E1510311A0, Qingdao, China). The analytical method and 3D-HPLC profile of the NYT extract have been reported previously [29].

### Animals

Six-week-old C57BL/6J male mice were purchased from Japan SLC (Shizuoka, Japan) and maintained under the following conditions: temperature, 23 ± 2 °C; relative humidity, 55 ± 10%, and a 12:12 light–dark (L:D) cycle with the lights on from 8:00 to 20:00. During the preliminary breeding period, the mice were provided breeding feed, CE-2 (CLEA Japan, Tokyo, Japan).

### NYT treatment in mice with NASH that were fed CDAHFD

Seven-week-old C57BL/6J male mice (weighing 19.3 ± 0.5 g) were divided into four groups (each group *n* = 8): normal diet group (normal group, CE-2), CDAHFD group (control group, A06071302; Research Diets, New Brunswick, NJ, USA), CDAHFD + 1000 mg/kg NYT group (NYT1000 group), and CDAHFD + 1500 mg/kg NYT group (NYT1500 group). Regarding NYT, a daily dose of 7.5 g (bulk extract 6.7 g) in humans (60 kg B.W.) is approximately 1500 mg/kg in mice (20 g B.W.) [30]. Based on this dosage, we divided the study into the low-dose 1000 mg/kg and equal-dose 1500 mg/kg groups. Given that CDAHFD reportedly induces NASH after 6 weeks of feeding [31], the duration of NYT administration was adjusted based on the time required to establish the NASH model. Subsequently, they were maintained for 6 weeks. An aqueous solution of NYT extract (0.1 ml per 10 g of mouse body weight) was orally administered once daily for 6 days a week to the NYT1000 and NYT1500 groups at 1000 and 1500 mg/kg of NYT, respectively. An equal quantity of distilled water was orally administered to the normal and control groups. The body weight of the mice was measured daily. On the last day of the experiment, the mice were subjected to laparotomy under anesthesia (isoflurane inhalation), and blood was collected from the abdominal inferior vena cava; the mice were subsequently euthanized. After blood collection, the liver, adipose tissue, and gastrocnemius muscle were harvested, and their wet weight was measured. Aspartate transaminase (AST), alanine transaminase (ALT), triglycerides, total cholesterol, and free fatty acid levels in the mice blood were measured using Transaminase CII Test Wako (FUJIFILM Wako Pure Chemical, Osaka, Japan), Triglyceride E-Test Kit (FUJIFILM Wako Pure Chemical), Cholesterol E-Test Wako Kit (FUJIFILM Wako Pure Chemical), LabAssay NEFA (FUJIFILM Wako Pure Chemical), and Synergy H1 Hybrid Multi-Mode Microplate Reader (BioTek Instruments, Tokyo, Japan).

### Metabolic, histopathological, and gene expression analyses

Lipid extraction from the liver and gastrocnemius muscle was performed using a partial modification of the Folch method [32]; 5 and 2 ml of a chloroform and methanol mixture (2:1) was added to the liver and gastrocnemius muscle tissue sample, respectively, and homogenization was performed under ice-cold conditions. The mixture was then allowed to stand for 10 min and centrifuged at 3000 rpm for 10 min. The organic layer was separated, the organic solvent was removed using nitrogen gas, and 10 µl of ethanol was added. Then, triglyceride and total cholesterol levels were measured in the samples. Hydroxyproline was measured using Total Collagen Assay (QuickZyme Biosciences, Leiden, The Netherlands) according to the manufacturer’s recommended protocol and Synergy H1 Hybrid Multi-Mode Microplate Reader (BioTek Instruments).

Liver tissue fragments were fixed with 4% paraformaldehyde, embedded in paraffin, sliced into 4 µm sections, stained with hematoxylin–eosin (HE), Masson’s trichrome (Muto Pure Chemicals, Tokyo, Japan), and Picro-Sirius red staining (Polysciences, Warrington, PA, USA), and observed using an Axio Observer inverted microscope (Carl Zeiss Microscopy GmbH, Munich, Germany). For Oil red O staining (Junsei Chemical, Tokyo, Japan), liver tissues soaked in 4% paraformaldehyde were embedded using Tissue-Tek O.C.T. Compound (Sakura Finetek Japan, Torrance, CA, USA) and sliced into 10 µm sections and stained. Specimens were visualized using the Axio Observer Inverted microscope. Each stained section (three random microscopic fields per section; magnification: 200×) was quantified using ImageJ Fiji Software (National Institutes of Health, Bethesda, MD, USA).

Total RNA was extracted from liver samples stored in RNAlater Stabilization Solution (Thermo Fisher Scientific, Waltham, MA, USA) using TRIzol Reagent (Thermo Fisher Scientific). cDNA synthesis was performed using ReverTra Ace qPCR RT Master Mix with gDNA Remover (TOYOBO, Osaka, Japan). After the addition of THUNDERBIRD SYBR qPCR Mix (TOYOBO) and primers (Table 2), the gene expression level was calculated according to the 2^–ΔΔCt^ method using StepOnePlus Real-Time PCR System (Thermo Fisher Scientific).

**Table 2:** Oligonucleotide primers used in real-time RT-PCR

Genes	Forward primers (5′→3′)	Reverse primers (5′→3′)
α-SMA	GTCCCAGACATCAGGGAGTAA	TCGGATACTTCAGCGT
TGF-β1	GGCACCGGAGAGCCCTGGATA	AATGTACAGCTGCCGCACACAGC
GAPDH	TCCTGTGGCATCCACGAAACT	GAAGCATTTGCGGTGGACGAT

α-SMA, α-smooth muscle actin; TGF-β1, transforming growth factor-β1; GAPDH, glyceraldehyde 3-phosphate dehydrogenase

### Locomotor activity analysis

Spontaneous locomotor activity of mice was measured 6 weeks after CDAHFD feeding using SUPERMEX PAT.P (Muromachi Kikai, Tokyo, Japan). The movement of mice was monitored using a passive infrared sensor and recorded using a digital counter. The mice were placed in plastic cages (30 × 20 × 13 cm; one mouse per cage). After acclimatization, spontaneous locomotor activity at night (12 h), which is the active phase of mice, was measured.

### Statistical analysis

Measured values were expressed as mean ± standard deviation or median [25% tile, 75% tile]. Statistical analyses were performed using EZR [33], a statistical software that extends the functionality of R (version 3.6.1), and R Commander. Significance was determined using the Dunnett’s or Steel test; *p* < 0.05 was considered significant.

## Results

### Effects of NYT on mice physiological characteristics

CDAHFD was given to the mice for 6 weeks to establish a NASH model [31]. After CDAHFD feeding, the control group showed significant weight loss and increased liver weight compared to the normal group, but no improvement was observed in the NYT1000 and NYT1500 groups (Table 3). Considering average daily food intake, there were no differences in the amount of food intake across the groups. The weight of white and brown adipose tissue substantially increased and tended to decrease (*p* = 0.06), respectively, in the control group compared with that of the normal group. The NYT1000 group showed a notable decrease and increase in white and brown adipose tissue weights, respectively, compared with that of the control group. Plasma biochemical analysis revealed changes in some metabolic markers due to CDAHFD feeding. However, no changes were induced by NYT treatment.

**Table 3:** Effect of Ninjin’yoeito (NYT) on physiological characteristics and plasma markers of mice with NASH

	Normal	NASH		
		Control	NYT1000	NYT1500
Initial body weight, g	19.4 ± 0.5	19.4 ± 0.5	19.2 ± 0.4	19.4 ± 0.4
Final body weight, g	23.7 ± 0.9**	21.4 ± 0.7	20.9 ± 1.1	21.0 ± 0.6
Average daily food intake, g/day/N	2.9 ± 0.3	3.0 ± 0.5	2.8 ± 0.5	2.6 ± 0.5
Liver weight/body weight, mg/g (× 10^−2^)	4.7 ± 0.2**	6.8 ± 0.7	7.0 ± 0.5	7.2 ± 0.4
WAT/body weight, mg/g (× 10^−2^)	3.3 ± 0.3*	4.1 ± 0.9	3.2 ± 0.5*	3.8 ± 0.8
BAT/body weight, mg/g (× 10^−2^)	0.32 ± 0.03	0.29 ± 0.01	0.33 ± 0.04*	0.32 ± 0.04
Plasma AST, IU/L	9.9 ± 2.4**	155.4 ± 79.7	117.4 ± 32.2	122.4 ± 26.0
Plasma ALT, IU/L	3.4 ± 0.6**	62.9 ± 12.5	61.5 ± 14.3	72.2 ± 14.5
Plasma triglyceride, mg/dL	68.5 ± 23.0	60.6 ± 19.1	54.3 ± 11.6	56.7 ± 13.2
Plasma total cholesterol, mg/dL	70.3 ± 19.1**	39.2 ± 7.8	40.3 ± 8.3	49.3 ± 10.8
Plasma non-esterified fatty acid, mEq/L	0.63 ± 0.26	0.70 ± 0.27	0.65 ± 0.18	0.61 ± 0.18

NASH, non-alcoholic steatohepatitis; NYT, Ninjin’yoeito; WAT, white adipose tissue; BAT, brown adipose tissue; AST, aspartate aminotransferase; ALT, alanine aminotransferase. Data are presented as mean ± standard deviation (*n* = 8). *p < 0.05 and **p < 0.01 vs. the control group using Dunnett’s test

### Effects of NYT on liver steatosis

**Fig. 1:**
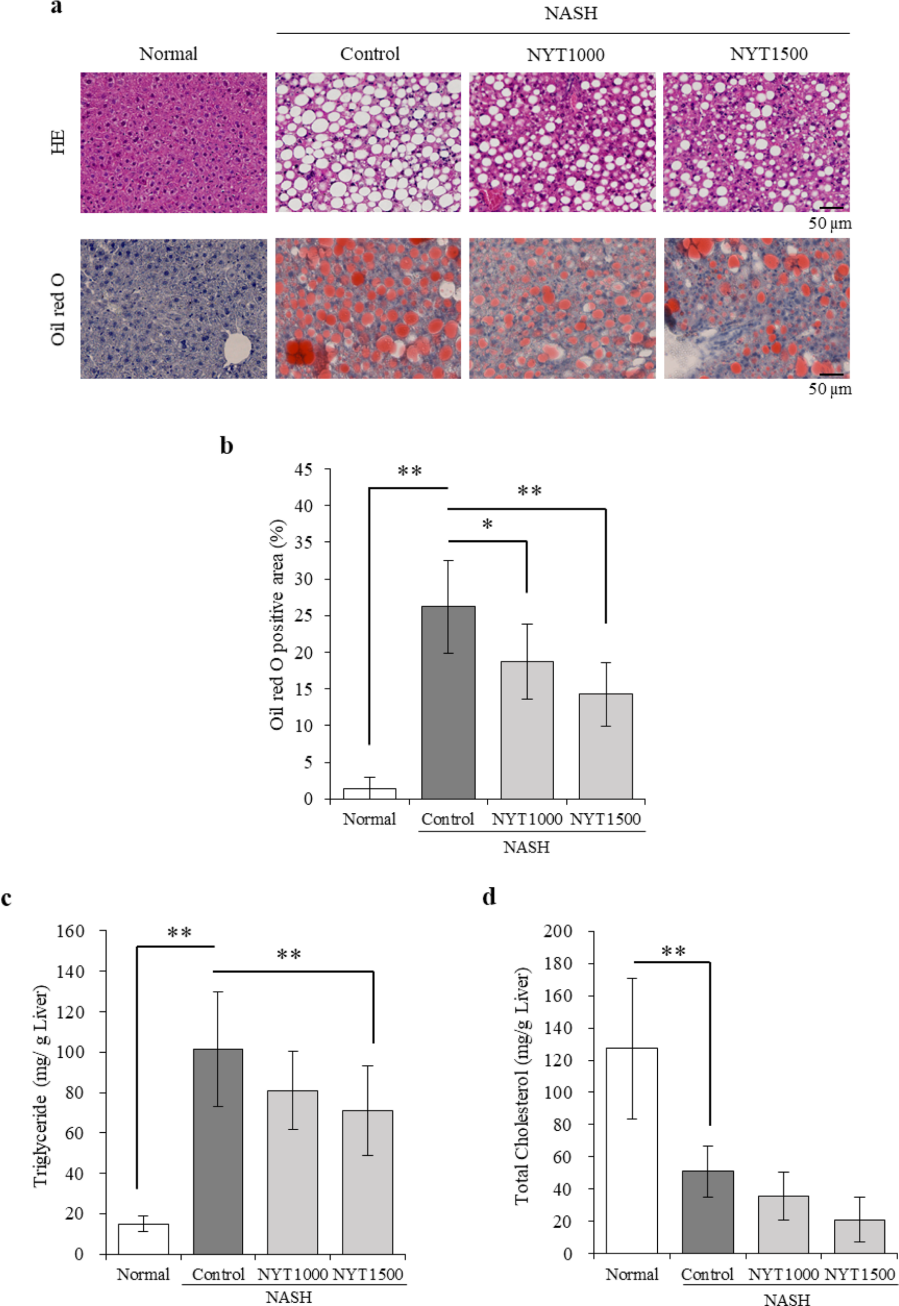
Effect of Ninjin’yoeito (NYT) on liver steatosis. **a** Hematoxylin–eosin (HE) and Oil red O staining of mouse livers to evaluate disease pathology (200×). **b** Percentage of Oil red O-positive area. **c** Hepatic triglycerides. **d** Hepatic total cholesterol. Data are presented as mean ± standard deviation (*n* = 6–8). *p < 0.05 and **p < 0.01 vs. the control group using Dunnett’s test

To test whether NYT suppressed steatosis in the liver, steatosis-related factors were studied and histopathological analysis was conducted. HE and Oil Red O staining revealed increased lipid droplets in the control group compared with the normal group (Fig. 1a and b). Lipid droplets decreased in the NYT1000 and NYT1500 groups compared with that in the control group. In addition, triglyceride levels were drastically increased in the control group compared with that in the normal group and decreased in the NYT1500 group compared with that in the control group (Fig. 1c). Total cholesterol content was substantially reduced in the control group compared with that in the normal group (Fig. 1d). No such difference was observed in the NYT1000 and NYT1500 groups compared with that in the control group.

### Effects of NYT on liver fibrosis

**Fig. 2:**
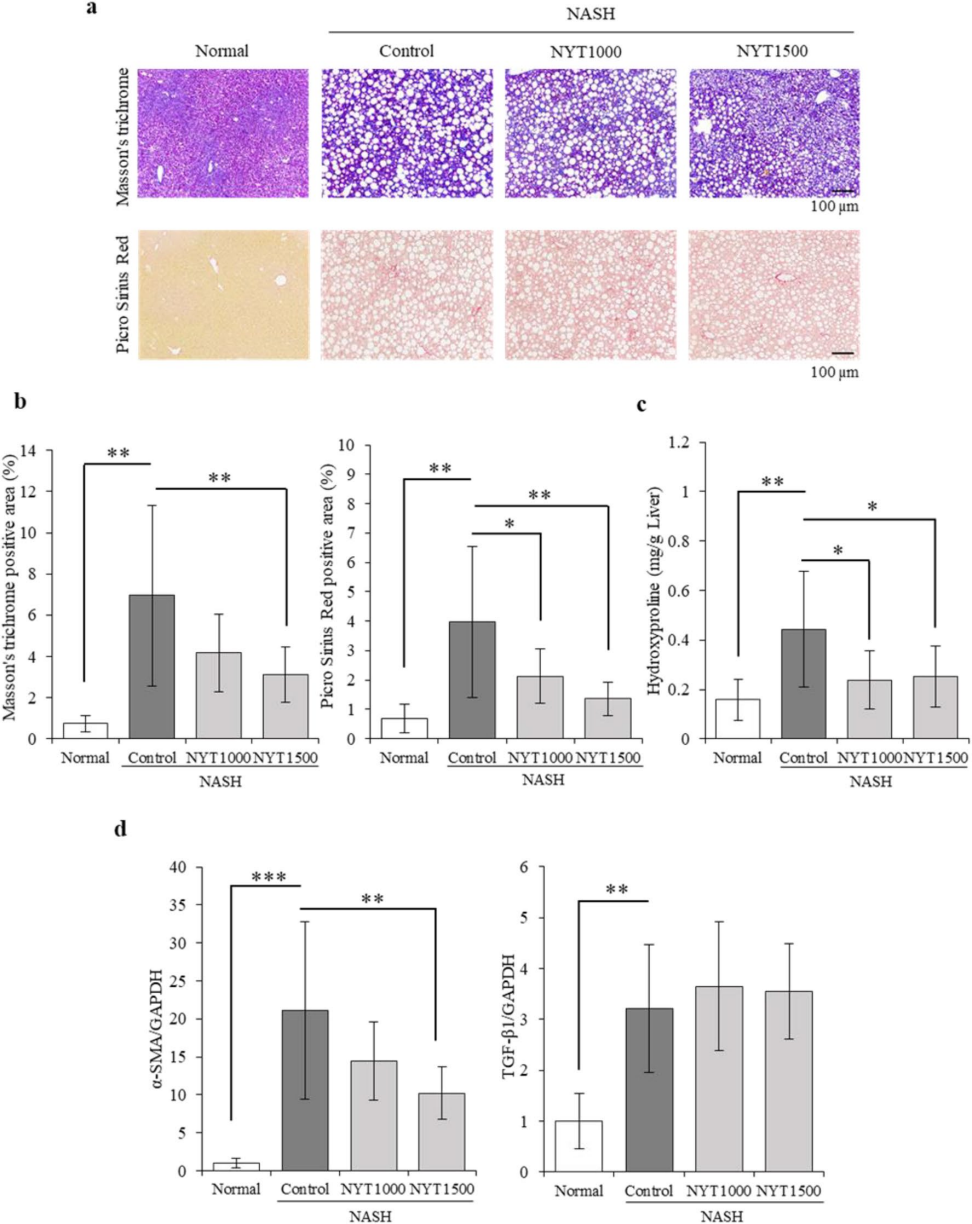
Effect of Ninjin’yoeito (NYT) on liver fibrosis. **a** Masson’s trichrome and Picro-Sirius red staining of the liver to evaluate disease pathology (200×). **b** The percentage of Masson’s trichrome-positive and Picro-Sirius red-positive areas. **c** Hepatic hydroxyproline. **d** Gene expression levels of α-smooth muscle actin (*α-SMA*) and transforming growth factor-β1 (*TGF-β1*) in the liver. Data are presented as mean ± standard deviation (*n* = 7–8). *p < 0.05 and **p < 0.01 vs. the control group using Dunnett’s test

Next, histopathological analysis was performed, and fibrosis-related factors and gene expression were analyzed in liver fibrosis. Masson’s trichrome and Picro-Sirius red staining showed a clear increase in collagen fibers in the control group (Fig. 2a and b). Specifically, liver fibrosis was observed in mice with NASH. In addition, collagen fibers in mice with NASH were reduced by NYT treatment. Hydroxyproline was increased in the control group compared with that in the normal group but decreased in the NYT1000 and NYT1500 groups compared with that in the control group (Fig. 2c).

The expression of α-smooth muscle actin (*α-SMA*), an activation marker of hepatic stellate cells, and transforming growth factor-β1 (*TGF-β1*), a promoter of hepatic fibrosis that binds to the receptor of hepatic stellate cells, was measured. The expression of *α-SMA* and *TGF-β1* was drastically increased in the control group compared with that in the normal group (Fig. 2d). In addition, the expression of *α-SMA* was substantially decreased in the NYT1500 group compared with that in the control group.

### Effects of NYT on spontaneous locomotor activity and skeletal muscle in mice with NASH

**Fig. 3:**
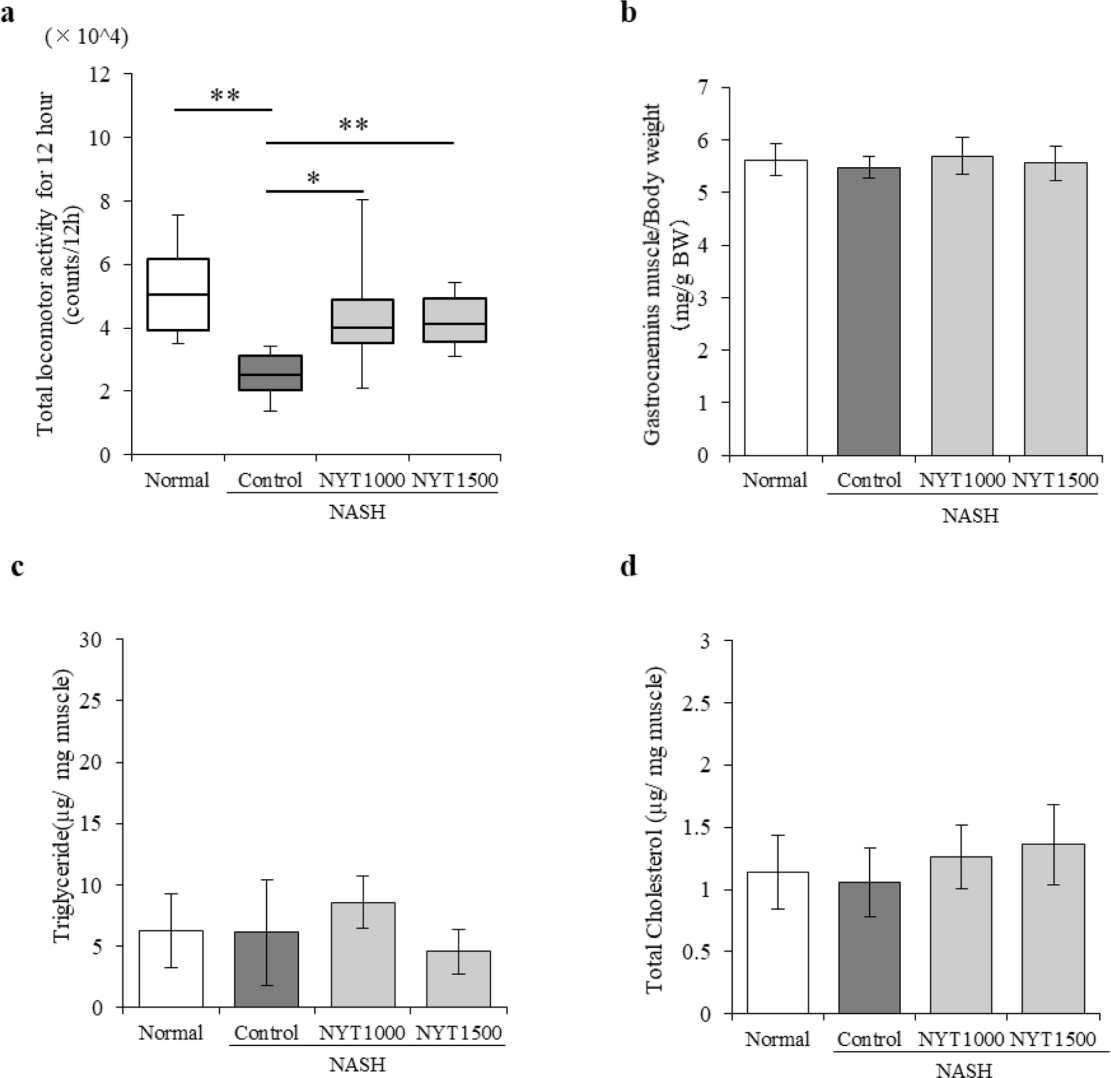
Effect of Ninjin’yoeito (NYT) on the locomotor activity and skeletal muscle weight of mice with non-alcoholic steatohepatitis (NASH). **a** Total locomotor activity during the dark phase (12 h) at 12 weeks. **b** Gastrocnemius muscle weight relative to body weight. **c** Triglycerides and **d** total cholesterol in the gastrocnemius muscle. Data are presented as **a** median [25% tile, 75% tile] and **b-d** mean ± standard deviation (*n* = 6–8). *p < 0.05 and **p < 0.01 vs. the control group using the Steel test

Measurement of spontaneous locomotor activity during the dark phase (12 h), which is the active phase in mice, revealed that compared with the normal group, the control group showed remarkably reduced locomotor activity (Fig. 3a). Specifically, a decrease in locomotor activity due to NASH was observed. However, locomotor activity increased in the NYT1000 and NYT1500 groups compared with that in the control group.

The weight and lipid content of the gastrocnemius muscle were measured after necropsy (Fig. 3b–d). No significant difference in the weight of gastrocnemius muscle was observed among the groups (Fig. 3b), and no locomotor disorders were observed. Furthermore, no significant differences in triglyceride and total cholesterol levels in the gastrocnemius muscle were observed among the groups (Fig. 3c and d).

## Discussion

Various approaches can be employed to establish a NASH model. Although a high-fat diet is a widely recognized method and deemed more appropriate for evaluating drug effects in clinically relevant conditions compared with other methods, the development of fibrosis is a time-dependent process, and the precise timing of early fibrosis remains uncertain. Given that the main objective of the current study was to confirm the protective effect of NYT against the progression of liver fibrosis, we opted for the CDAHFD model, in which the initial stage of fibrosis can be observed at 6 weeks of feeding [31].

In this study, hepatic steatosis, hepatic fibrosis, and decreased locomotor activity during the active phase were confirmed in mice with NASH that were fed CDAHFD, which closely mimic the pathology of NASH in the human liver [31, 34]. Furthermore, the oral administration of NYT protected against the pathological characteristics of NASH in mice. However, it should be noted that CDAHFD-induced NASH is different from human NASH in that it disables intrahepatic triglyceride metabolism, decreasing triglyceride and cholesterol levels in the blood [31, 35]. Moreover, because pharmacotherapy for NASH is not well established and the mechanism of NYT in the treatment of NASH was largely unknown, a positive control was not established in this study.

The protective effect of NYT-induced hepatic triglyceride accumulation led to the amelioration of liver steatosis. Triglycerides are known to accumulate in the liver of NASH mice [34], and NYT suppressed their accumulation. Cholesterol content in the liver was decreased by CDAHFD feeding, as previously reported [35]. NYT had no effect on liver cholesterol. How NYT affects hepatic lipid incretion, de novo lipogenesis, β-oxidation, and very low-density lipoprotein-TG excretion remains unclear. Poria Cocos, Atractylodes Rhizome, Ginseng, and their constituent herbs of NYT increase the expression of peroxisome proliferator-activated receptor α (PPARα) [36–38]. In addition, ginsenoside Rb2, a component of ginseng, induces Sirtuin1 [39]. NYT has been reported to act on peroxisome proliferator-activated receptor gamma coactivator 1α(PGC-1α), which is involved in the expression of PPARα in skeletal muscle [40] and may suppress fat accumulation by promoting β-oxidation of fatty acids through increasing the expression of PPARα. Therefore, this drug may inhibit fat accumulation by promoting β-oxidation of fatty acids through enhancing the expression of PPARα.

Activated hepatic stellate cells produce collagen type I, which is the main cause of fibrosis [41–43]. Given that hepatic stellate cells are deeply involved in liver fibrosis in NASH [1, 44], the same may be true for the mouse model in this study. The levels of *α-SMA*, an activation marker of hepatic stellate cells [45], and *TGF-β1*, a promoter of hepatic fibrosis that binds to the receptor of hepatic stellate cells, were measured. As a reduction in *α-SMA* expression was observed, the antifibrotic action by NYT may be accompanied by the suppression of hepatic stellate cell activation. The activation of hepatic stellate cells involves various pathways such as the TGF-β/SMAD, Wnt, and Gas6/Axl signaling pathways [46, 47], and the development of hepatic fibrosis is very complex. Of these pathways, NYT does not affect *TGF-β1* expression, suggesting that it may be involved in signaling pathways other than *TGF-β1*.

Skeletal muscle atrophy has been reported in NASH/NAFLD [48]. Therefore, in this study, muscular atrophy in the legs is assumed to be a cause of decreased locomotor activity during the active phase. However, no change in weight of the gastrocnemius muscle, an important lower limb muscle, was observed regardless of CDAHFD feeding or NYT treatment. In clinical practice, a relative decrease in skeletal muscle weight associated with fat accumulation in patients with NAFLD has been reported [49]; however, no change in lipid content was observed in the skeletal muscles of the mice in this study. Since triglyceride secretion from the liver to the blood vessels is suppressed by CDAHFD, fat accumulation may not have had an impact on the weight of the skeletal muscle. Therefore, from the results of skeletal muscle weight and fat content, it can be inferred that locomotor dysfunction was not involved in the decrease in locomotor activity. However, in some reports, decreased locomotor activity has been used as a surrogate marker for fatigue behavior [26–28]. Fatigue behavior with liver lesions has been reported in chronic liver diseases such as NASH [21–23]. Consequently, fatigue may be involved in the reduced locomotor activity of the mice in this study. Neuroinflammation in the brain, which is mediated by inflammation of the liver, is thought to be a cause of fatigue in NASH [23, 50–52]. NYT has been shown to be effective against fatigue and malaise [13, 15], improve liver lesions, and exert anti-inflammatory effects [20]. Therefore, the alleviation of locomotor activity reduction by NYT may involve a reduction of fatigue through the amelioration of liver lesions and inflammation. Future studies should elucidate the effectiveness of NYT on fatigue.

In this study, NYT was shown to improve hepatic steatosis, hepatic fibrosis, and spontaneous locomotor activity reduction during the active phase in NASH. In addition, considering that mice with NASH fed a CDAHFD are regarded to exhibit liver pathology closely resembling human NASH [31, 34], NYT holds promise as a potential therapeutic option for NASH treatment.

## Data Availability

All datasets generated during this study are included in the article.
